# Complete Genome Sequence of Nitrilotriacetate-Degrading *Aminobacter aminovorans* KCTC 2477^T^

**DOI:** 10.1128/genomeA.01363-16

**Published:** 2016-12-08

**Authors:** Sang-Heon Lee, Hanna Choe, Arshan Nasir, Doo-Sang Park, Kyung Mo Kim

**Affiliations:** a Biological Resource Center, Korea Research Institute of Bioscience and Biotechnology, Jeongeup, Republic of Korea; b Department of Polar Sciences, University of Science and Technology, Daejeon, Republic of Korea; c Division of Polar Life Sciences, Korea Polar Research Institute, Incheon, Republic of Korea; d Department of Biosciences, COMSATS Institute of Information Technology, Islamabad, Pakistan

## Abstract

*Aminobacter aminovorans* is a Gram-negative, pleomorphic rod-shaped, flagellated, and obligately aerobic bacterium that was isolated from soil. Here, we report the complete genome sequence of *A. aminovorans* KCTC 2477^T^, which degrades nitrilotriacetate-metal complexes and iminodiacetate, a metabolic intermediate of nitrilotriacetate.

## GENOME ANNOUNCEMENT

*Aminobacter aminovorans* is a Gram-negative, pleomorphic rod-shaped, flagellated, and obligately aerobic bacterium which is commonly found in soil (1). The type strain of *A. aminovorans* (KCTC 2477) was reported to aerobically degrade nitrilotriacetate (NTA), its metal ion complexes, and iminodiacetate (IDA), which is a metabolic intermediate of NTA (2). Therefore, *A. aminovorans* is regarded as a promising candidate for the bioremediation of NTA and for reducing the mobility of NTA-metal complexes. Despite its industrial importance, the complete genome sequence and associated information of strain KCTC 2477^T^ remain hitherto unavailable.

Strain KCTC 2477^T^ was grown for 3 days at 30°C on Nutrient agar. Genomic DNA was extracted using the i-genomic BYF minikit (iNtRON Biotechnology, Republic of Korea), according to the manufacturer’s protocols. Genome sequencing was performed using PacBio RSII single-molecule real-time (SMRT) sequencing technology (Pacific Biosciences, Menlo Park, CA, USA). A 20-kb insert SMRTbell library was constructed and sequenced using SMRT cell with P6-C4 chemistry, yielding 144-fold average genome coverage. *De novo* assembly of 86,158 reads representing 13,584 nucleotides on average (1,170,389,267 bp in total) was conducted using the Hierarchical Genome Assembly Process (HGAP) pipeline of SMRT Analysis version 2.3.0 (3). Next, protein-coding genes were searched by Prodigal version 2.6.1 (4). The predicted coding sequences (CDSs) were subjected to a BLAST search against the UniProt, Pfam, and COG databases. Signal peptides and transmembrane helices were detected using SignalP version 4.1 and TMHMM version 2.0. rRNA, tRNA, and other miscellaneous features were predicted using RNAmmer version 1.2, tRNAscan-SE version 1.21, and Rfam version 12.0. References for annotations of protein-coding and noncoding regions can be found in a study by Lee et al. (5).

The complete genome of *A. aminovorans* KCTC 2477^T^, with 63.14% G+C content, is made up of one circular chromosome of 5,623,946 bp and four circular plasmids (pAA01, pAA02, pAA03, and pAA04) of 1,266,780 bp. The coding regions cover 87.81% of the genome (6,050,925 bp), encoding a total of 6,613 proteins. Signal peptides and transmembrane helices were detected in 577 (8.73%) and 1,517 (22.94%) protein-coding genes, respectively.

The KCTC 2477^T^ genome harbors several genes directly related to NTA degradation. For example, the existence of an *nta* gene cluster (e.g., locus tag AA2016_6031 to AA2016_6033), *nta* operon transcriptional regulator (e.g., AA2016_6031), nitrilotriacetate monooxygenase-coding genes (AA2016_6032 and AA2016_6425), and NADH:flavin mononucleotide (NADH:FMN) oxidoreductase-coding genes (AA2016_6033 and AA2016_6429) highlights the potential of KCTC 2477^T^ to degrade NTA into metabolic intermediates, such as IDA (6). Specifically, NTA monooxygenase uses FMNH_2_ and O_2_ to oxidize NTA, and NADH:flavin mononucleotide oxidoreductase provides FMNH_2_ for NTA oxidation. Therefore, the genome data of KCTC 2477^T^ will serve as an invaluable resource to understand the complete metabolic pathway of NTA degradation, eventually leading to improved efforts toward the neutralization of NTA and its associated complexes from the environment.

### Accession number(s).

The complete genome sequence has been deposited at GenBank/EMBL/DDBJ under the accession numbers CP015005 (chromosome), CP015006 (plasmid pAA01), CP015007 (pAA02), CP015008 (pAA03), and CP015009 (pAA04). This strain is available from the Korean Collection for Type Cultures (Jeongeup, Republic of Korea) under the accession no. KCTC 2477.
